# Some new or poorly-known Zephroniidae (Diplopoda, Sphaerotheriida) from Vietnam

**DOI:** 10.3897/zookeys.930.47742

**Published:** 2020-04-28

**Authors:** Irina Semenyuk, Sergei I. Golovatch, Thomas Wesener

**Affiliations:** 1 A. N. Severtsov Institute for Problems of Ecology and Evolution, Russian Academy of Sciences, Leninsky pr. 33, Moscow 119071, Russia A. N. Severtsov Institute for Problems of Ecology and Evolution, Russian Academy of Sciences Moscow Russia; 2 Joint Russian-Vietnamese Tropical Center, Street 3 Thang 2, 3, Q10, Ho Chi Minh City, Vietnam oint Russian-Vietnamese Tropical Center Ho Chi Minh Vietnam; 3 Zoological Research Museum Alexander Koenig (ZFMK), Leibniz Institute for Animal Biodiversity, Adenauerallee 160, D-53113, Bonn, Germany Zoological Research Museum Alexander Koenig Bonn Germany

**Keywords:** biological observations, giant pill-millipede, *
Sphaerobelum
*, *
Sphaeropoeus
*, taxonomy

## Abstract

Three new species of the giant pill-millipede family Zephroniidae are described from southern Vietnam: *Sphaerobelum pumatense***sp. nov.**, *Sphaeropoeus honbaensis***sp. nov.** and *Sphaeropoeus bidoupensis***sp. nov.** Two species, *Sphaerobelum bicorne* Attems, 1938 and *Sphaeropoeus maculatus* (Verhoeff, 1924), are redescribed, the former from new material, the latter from type material with lectotype designation. A new transfer is proposed: *Zephronia manca* Attems, 1936, to the genus *Sphaeropoeus* Brandt, 1833, giving the new combination, *Sphaeropoeus manca* (Attems, 1936) **comb. nov.**

## Introduction

The giant pill-millipede fauna (order Sphaerotheriida) of Southeast Asia is dominated by members of the family Zephroniidae (Wesener 2016a). This family is the only one in the order that occurs in Indochina. According to the latest review (Semenyuk et al. 2018), the Vietnamese fauna is particularly rich in zephroniids and currently contains six species of *Sphaerobelum* Verhoeff, 1924, five species of *Zephronia* Gray, 1832, three species of *Prionobelum* Verhoeff, 1924, and one species each in *Cryxus* Leach, 1814 and *Sphaeropoeus* Brandt, 1833.

The present paper puts on record another three new zephroniid species from Vietnam, provides redescriptions of two previously described species, and establishes a new combination.

## Material and methods

### Collecting, dissecting and drawing

Field work of author IS was conducted in accordance with Agreement 37/HD for the scientific cooperation between the Joint Russian-Vietnamese Tropical Centre and the Bidoup Nui Ba National Park and Hon Ba Nature Reserve, according to Agreement 1700/UBND.VX for the Pu Mat National Park, and to Agreement 308/SNgV-LS for the Song Thanh National Park. The Animal Care and Use Protocol Review No. 1723018 was strictly followed.

Material was collected in the Bidoup Nui Ba National Park during three field trips: 5–16 January, 12–21 June and 22–27 November 2018. The field work in the Hon Ba Nature Reserve was carried out from 23–29 June 2018, that in the Pu Mat National Park from 11–24 April 2018, while that in the Song Thanh National Park was from 23 April to 11 May 2019. Millipedes were obtained in forest or rural habitats by hand-sorting the leaf litter, visual spotting on open places, and through the examination of spaces under logs and stones. Collecting was performed in daylight, as well as at night. Ecological and behavioral data were recorded while collecting. Pictures of living animals were taken with a Panasonic DMC-TZ80 - LUMIX Digital Camera. Animals were preserved in 75% ethanol.

For illustrations, the 9^th^ legs and both anterior and posterior telopods were removed from males with forceps. Female vulvae were dissected from leg-pair 2 on one side of the body (not dissected in one species). Antennae were examined for all species where females were available, and male antennae were illustrated without removing the head. An Olympus SZ61 stereo microscope was used for observation and capturing images for the line drawings; sketches were scanned with a CanoScan Lide 60 scanner and then edited with Corel Photo-Paint X5 software. The terminology of morphological characters follows Wesener and Sierwald (2005) and Wesener (2016a). New specimens, including types, were shared between the collections of the Zoological Museum, State University of Moscow, Russia (**ZMUM**) and the Zoological Research Museum A. Koenig, Bonn, Germany (**ZFMK**), as indicated below. Types of existing species were borrowed and/or used from the collections of the Natural History Museum in Vienna (**NHMW**) and the Hungarian Natural History Museum in Budapest (**NHMB**).

### Scanning electron microscopy (SEM)

Left antennae, endoterga and terga were dissected. The samples were cleaned and dehydrated via an ethanol series (2× 96%, 3× 100%) prior to mounting on aluminum SEM stubs. The samples were coated with gold for 240 seconds in a sputter coater. SEM images were then taken using a Supra VR 300VP (Carl Zeiss AG, Oberkochen, Germany) scanning electron microscope utilizing SmartSEM V05.00 software based at the ZFMK. Dry-coated SEM material was then removed after study from the stubs and returned to alcohol. All images were later edited using Adobe Photoshop CS2 and assembled into plates with Adobe Illustrator CS2 (San Jose, USA).

## Results

### Taxonomy

**Class Diplopoda de Blainville in Gervais, 1844**

**Order Sphaerotheriida Brandt, 1833**

**Family Zephroniidae Gray in Jones, 1843**

### Genus *Sphaerobelum* Verhoeff, 1924

See Wesener (2016a) for a recent diagnosis of the species, and Wesener (2019) for a key.

#### Sphaerobelum bicorne

Taxon classificationAnimaliaDiplopodaZephroniidae

Attems, 1938

43FB7CBE-A82A-5868-AB1A-EFF92A6F435B

**Figure 1.**
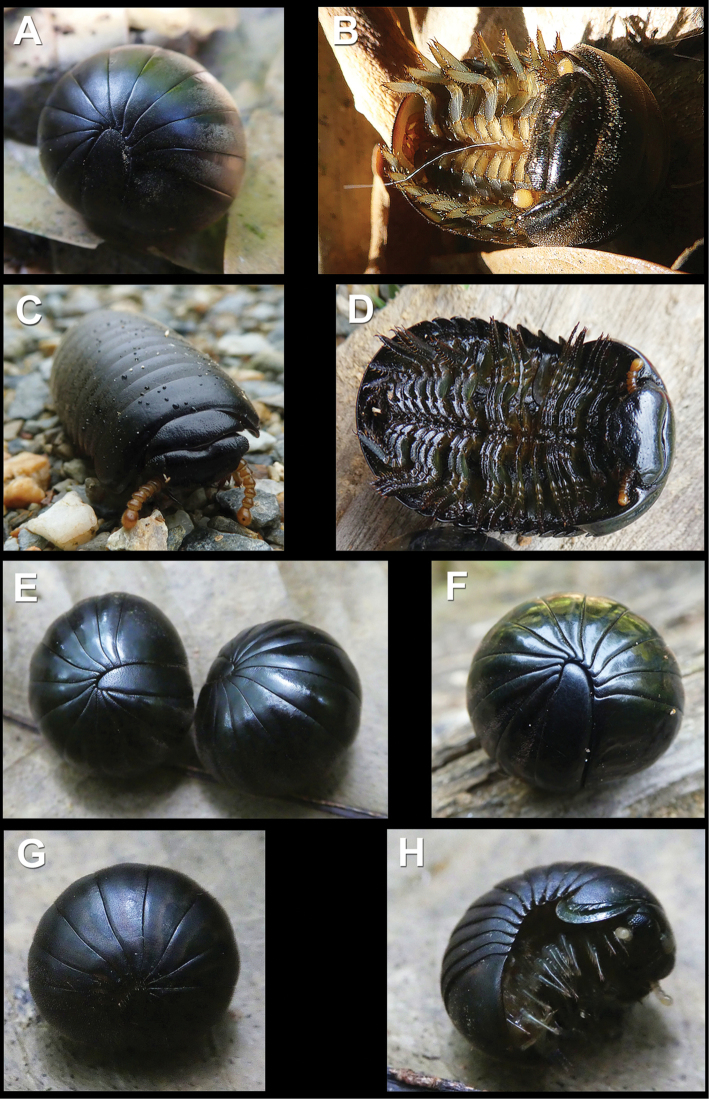
Live *Sphaerobelum pumatense* sp. nov. (**A–C**) and *Sphaerobelum bicorne* Attems, 1938 (**D–H**) in the field **A–C** adult, enrolled, ventral view and walking, respectively **D–F** adults, ventral view, and enrolled, respectively **G, H** juvenile, enrolled and unrolling, respectively. Pictures taken not to scale.

Figs 1A–C , 3A , 4A , 5

Sphaerobelum bicorne Attems, 1938: 200; Enghoff et al. 2004: 32 (list); Jeekel 2001: 17 (list); Wongthamwanich et al. 2012: 34 (key); Wesener 2019: 211 (key).

##### Material examined.

1 ♂, 1 ♀ (ZFMK MYR8860), 2 ♂, 5 ♀, 2 juv. (ZMUM Rd 4636), Vietnam, Quang Nam Prov., Song Thanh National Park, 15°33'N, 107°23'E, 1000 m a.s.l., tropical forest in a narrow river valley, on forest floor (♂) and in leaf litter (♀), daytime, V.2019, I. I. Semenyuk leg. ***Syntypes*** 1 ♂, 1 ♀ (NHMW 2196), Vietnam, South Annam (Tourane = Da Nang), Ba Na Hills, C. Dawydoff leg. Studied only from numerous photographs by author TW.

##### New diagnosis.

*Sphaerobelum bicorne* belongs to the group of congeners in which the mesal margin of the femur is extended into several teeth. *Sphaerobelum bicorne* shares only with *S. bolavensis* Wesener, 2019, from Laos (Wesener 2019), the presence of such an extension in the apical part of the femur, but differs from the latter species in several characters: ♀ operculum projecting into two conspicuous processes (Fig. 5H) (vs. one process in *S. bolavensis*), telopoditomere 4 of posterior telopod straight, apically with a recessed hook (Fig. 5A, B) (vs. no hook in *S. bolavensis*), and locking carina of anal shield long (vs. short in *S. bolavensis*).

##### Redescription (mostly based on ZFMK material).

***Body length***: ♂ length ca. 40.9 mm, width of thoracic shield 21.3 mm, of tergite 8, 21.9 mm (= broadest), height of thoracic shield, 12.1 mm, of tergite 8, 13.3 mm (= highest); ♀ length ca. 42.7 mm, width of thoracic shield, 23.5 mm, of tergite 7, 24.3 mm (= broadest), height of thoracic shield 11.9 mm, of tergite 8, 15.1 mm (= highest). ZMUM adults 18 (♂) to 23 mm wide (♀). ***Coloration***: both in vivo and in vitro, after several months of preservation in ethanol, uniformly black to blackish, shining. Head and collum also black. Antennae orange, legs in life mainly blackish as well (Fig. 1D–F), but in alcohol dark olive, usually with several basal segments and tarsi or their distal halves orange, only juveniles a little lighter, dark brown to blackish, some with very vague variegated tergal patterns (Fig. 1G, H). ***Head***: eyes with >70 ocelli. Aberrant ocellus located inside antennal groove. Antennae short (Fig. 5G), with rounded joints, protruding posteriorly to leg-pair 3. All antennomeres densely pubescent, sensilla basiconica surrounding apical disc. Shape of antennae sexually dimorphic, cylindrical in ♀, thickened, apically broadened and slightly flattened in ♂. Apical disc with ca. 74/76 (♂) or 56/51 (♀) apical cones, respectively. Apical cones typical of Diplopoda. Organ of Tömösváry located inside antennal groove. ***Gnathochilarium***: structure typical of the order. Palpi with sensory cones arranged in clusters. ***Mandibles***: not dissected. ***Stigmatic plates***: first stigmatic plate broadly rounded, apex clearly rounded, weakly curved towards coxa 1. ***Laterotergites***: laterotergite 1 strongly projecting into a well-rounded tip. Laterotergite 2 well-rounded, like following laterotergites. ***Collum***: with a glabrous surface, margins with few isolated setae. ***Thoracic shield***: surface glabrous like tergites, setae only in grooves. Shallow grooves beset with numerous long setae, sloping towards groove with 5 or 6 continuous lateral and posterior keels. ***Tergites***: surface of anterior half of tergites setose, with very small setae and small pits, posterior half of tergite smooth (Fig. 4A). Tips of midbody paratergites projecting posteriorly. ***Endotergum***: inner section lacking any spines or setae. Middle area with a single row of large, dense, elliptical, cuticular impressions. Distance between impressions shorter than half their diameter. Apically, 3–4 dense rows of long marginal bristles, tips of longest setae clearly protruding beyond tergal margin (Fig. 3A). Bristles not smooth, but with numerous small spinicles. ***Anal shield***: large, sexually dimorphic: in ♀ well-rounded, in ♂ weakly bell-shaped. Surface in ♀ only in anterior half, in ♂ completely covered with tiny setae. Underside with a single, long, black, locking carina, this being slightly longer than width of last laterotergite, locking carina located close to last laterotergite. ***Legs***: leg 1 with 4, leg 2 with 5, leg 3 with 6 or 7 ventral spines. First two leg-pairs each without an apical spine. Leg-pairs 4–21 with 7–9 ventral spines and one dorso-apical spine (Fig. 5F). In leg 9, femur 1.7, tarsus 3.8 times longer than wide (Fig. 5F). All podomeres densely setose. Coxa with a large and well-rounded process. Coxa process sharp in legs 1 and 2. Prefemur apico-mesally with a weak projection. Femur in apical part extended mesally into a dentate margin featuring 4–6 teeth. ***Female sexual characters***: vulva large, covering 2/3 of coxa, extending mesally to anterior half of prefemur (Fig. 5H). Operculum centrally deeply recessed, apical margin projecting into two rounded lobes, 2–3 times as high as remaining operculum (Fig. 5H). Subanal plate well-rounded, almost circular. ***Male sexual characters***: gonopore covered with a single, undivided, circular, sclerotized plate. ***Anterior telopods*** (Fig. 5C–E): consisting of only 3 telopoditomeres distal to syncoxite, telopoditomeres 3 and 4 partly fused. Telopoditomere 1 cylindrical, slightly longer than wide. Telopoditomere 2 large, without process as long as telopoditomere 3. Process of telopoditomere 2 located posteriorly, visible in anterior view. Process slender, projecting to 2/3 of telopoditomere 3, conspicuously curved, with an almost sharp apex. Telopoditomere 3 massive, cylindrical, straight, apically slightly tapering. Posterior side with a black sclerotized spot and a small, triangular spine. Telopoditomere 1 in apical view covered with long setae. In posterior view all telopoditomeres setose. ***Posterior telopods*** (Fig. 5A, B): telopoditomere 1 large and cylindrical, twice as long as wide, reaching the length of telopoditomere 3. Immovable finger (process of telopoditomere 2) shorter than movable finger, consisting of telopoditomeres 3 and 4. Immovable finger with a characteristic, distally swollen apex, clearly rounded, apex therefore wider as base, projecting especially strongly at lower margin. Telopoditomere 3 rectangular, clearly rounded, with a sharp process directed towards immovable finger. Telopoditomere 4 as long as, but slightly more slender than, telopoditomere 3, 2.5 times longer than wide, apically weakly tapering, with a tiny curved hook directed towards immovable finger. Telopoditomere 1 on both sides covered with setae, remaining telopoditomeres in posterior view almost glabrous, in anterior view with few isolated setae except for immovable finger which is more densely setose.

##### Remarks.

In the field, these millipedes were found in a very wide range of habitats, from 700 to 1200 m a.s.l., including extremely humid forest on river banks and in valleys with abundant *Cyathea* sp. tree ferns, on sandy soils and in sparse leaf litter; on hill slopes covered with rich broadleaved tropical forest and a thick leaf litter layer; as well as on very dry ridges and interfluves with broadleaved forest with admixtures of coniferous trees, and in open places colonized by *Dicranopteris* sp. ferns and *Melastoma* sp. bushes. A similar number of males and females were recorded during the expedition (30 adult individuals in total). Two-thirds of the females were hidden in leaf litter inside their “living chambers”, the remaining were spotted walking on the forest floor. All males were likewise walking on the forest floor, most probably searching for mates. The few recorded juveniles of both sexes were hidden in leaf litter. During the expedition, the day temperature on the leaf litter surface averaged 24 °C, compared to 19 °C in the night, with occasional nights when the temperature dropped down to 17.5 °C. Heavy, but rather short showers took place almost every day, quite often also with fogs in the evening. The abundance of the millipedes did not change drastically under rains, only slightly decreasing on non-rainy days in open habitats.

According to local knowledge, April is the driest month in the Park, while the rest of the year is extremely humid. Surprisingly, we noticed the lack of Diplopoda during the expedition with very little millipede activity, but *S. bicorne* was abundant at different age stadia. It may be a strategy for avoiding competition with other millipede species, as *Sphaerobelum* is a quite robust and well-protected diplopod capable of surviving difficult conditions.

**Figure 2.**
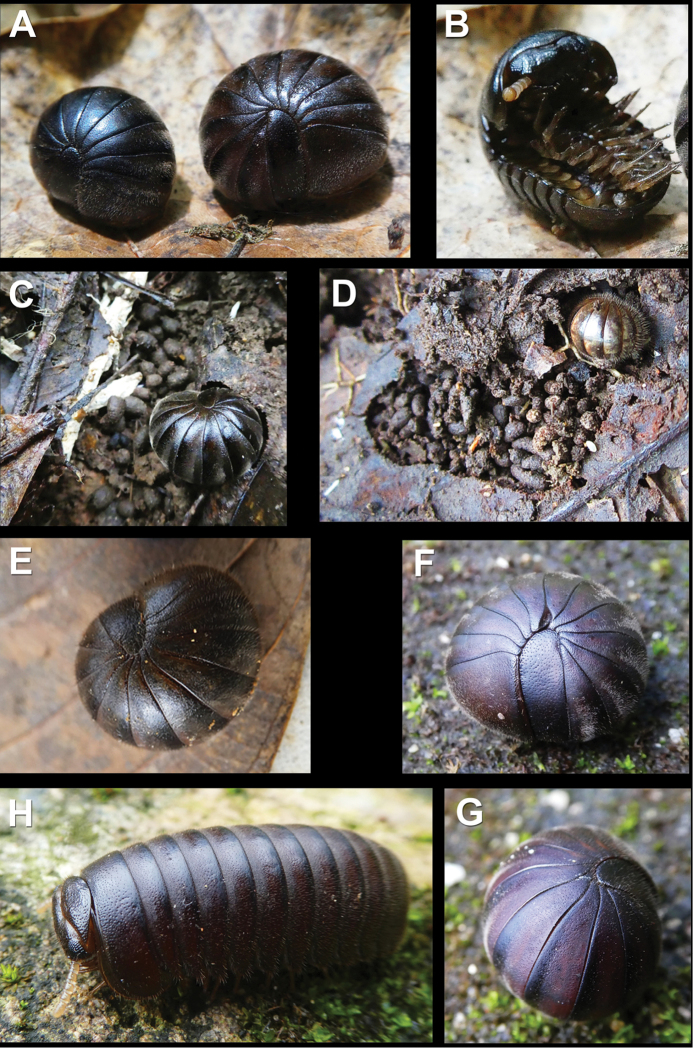
Live *Sphaeropoeus bidoupensis* sp. nov. (**A–E**) and *Sphaeropoeus honbaensis* sp. nov. (**F–H**) in the field **A** enrolled male (left) and female (right) **B** adult, ventral view **C** adult and its excrements in a “living room” in leaf litter **D** early instar juvenile enrolled in its “living room”, with surrounding dead leaves eaten and excrement used to shape the room **E** enrolled middle-sized instar juvenile **F–H** adult, enrolled and walking, respectively. Pictures taken not to scale.

#### Sphaerobelum pumatense sp. nov.

Taxon classificationAnimaliaDiplopodaZephroniidae

4D4EE2CF-60CE-51CD-92C5-8B1DB6EE3685

http://zoobank.org/180236D4-C6AD-4682-B2C2-911BE419F552

Figs 1A–C , 3B , 6

##### Material examined.

***Holotype*** ♂ (ZMUM Rd 4647), Vietnam, Nghe An Prov., Pu Mat National Park, 18°56'N, 104°38'E, 200 m a.s.l., mixed tropical forest on steep slopes, on forest floor, daytime, IV.2018, I.I. Semenyuk leg. ***Paratypes*** 1 ♂, 1 ♀ (ZMUM Rd 4632), 1 ♂, 2 ♀ (ZMUM Rd 4648), 1 ♂, 1 ♀ (ZFMK MYR8942), same data as holotype.

##### Diagnosis.

*Sphaerobelum pumatense* sp. nov. belongs to the group of congeners in which the mesal margin of the femur is extended into several teeth (Fig. 6F). In addition, *S. pumatense* sp. nov. shares only with *S. spinatum* Wesener, 2019 and *S. nigrum* Wesener, 2019, both from Laos, and with *S. cattiense*Semenyuk et al., 2018 and *S. konkakinhense*Semenyuk et al., 2018, both from Vietnam, a straight telopoditomere 4 of the posterior telopod (Fig. 6A, B). *Sphaerobelum pumatense* sp. nov. differs in several unique characters from the four other species: vulval operculum not projecting mesally into a strong tip (Fig. 6H); telopoditomere 4 of posterior telopods exceptionally slender, >5× as long as wide (Fig. 6A, B).

**Figure 3.**
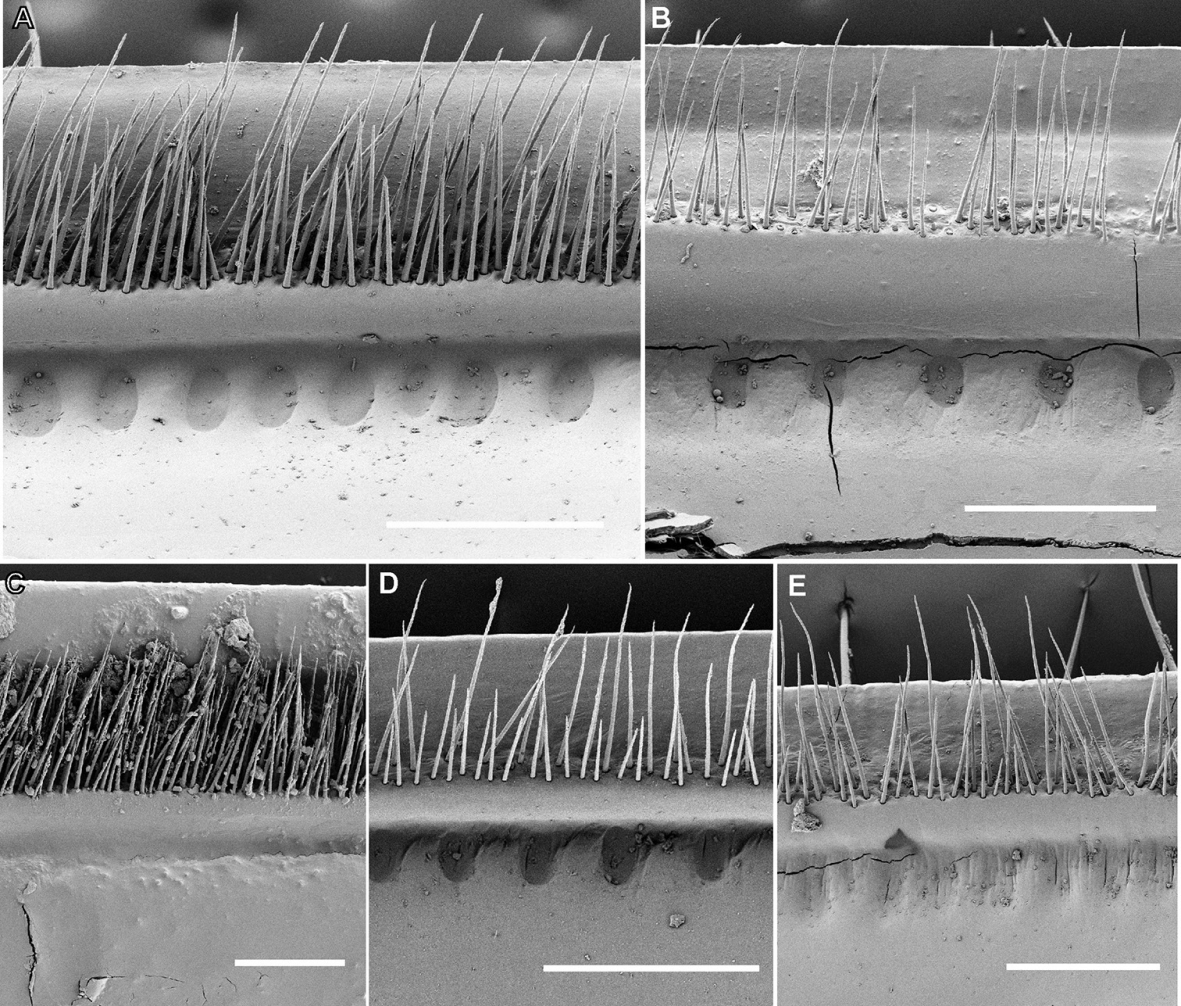
Endoterga of midbody segments, SEM micrographs. **A***Sphaerobelum bicorne* Attems, 1938, ♂ (ZFMK) **B***Sphaerobelum pumatense* sp. nov., ♂ holotype **C***Sphaeropoeus maculatus* (Verhoeff, 1924), ♂ lectotype **D***Sphaeropoeus bidoupensis* sp. nov., ♂ holotype **E***Sphaeropoeus honbaensis* sp. nov., ♂ holotype. Scale bars: 0.2 mm.

##### Description.

***Body length***: holotype ♂ length ca. 27.2 mm, width of thoracic shield 15.61 mm, of tergite 7 (= broadest), 15.5 mm, height of thoracic shield, 7.9 mm, of tergite 7 (= highest), 8.2 mm; ♀ length ca. 34.8 mm, width of thoracic shield, 17.2 mm, of tergite 7, 17.9 mm (= broadest), height of thoracic shield, 9.5 mm, of tergite 7, 9.9 mm (= highest). Width of paratypes 14–17 (♂) or 15–17 mm (♀). ***Coloration***: both in vivo and in vitro, after >1.5 years of preservation in ethanol, similar, uniformly dark blackish brown, dark chestnut, dark grey-brown or dark reddish brown, in alcohol with posterior margin usually dark brown, paratergite depressions, groove of thoracic shield and legs dark olive to olive-brown, but several basal podomeres and distal halves of tarsi considerably lighter, orange. Antennae blackish to dark brown, but antennomeres 5 and 6 lighter, light brown to nearly orange. Tegument mosly dull to poorly shining (Fig. 1A–C). ***Head***: eyes with >75 ommatidia. Aberrant ocellus located inside antennal groove. Antennae short (Fig. 6G), with rounded joints, protruding posteriorly to leg-pair 3. Lengths of antennomeres: 1 = 2 = 3 = 4 = 5 << 6. All antennomeres densely pubescent, sensilla basiconica surrounding apical disc. Shape of antennae sexually dimorphic, cylindrical in ♀, thickened, apically broadened and flattened in ♂. Apical disc with ca. 63/67 (♂) or 48/45 (♀) apical cones, respectively. Organ of Tömösváry located inside antennal groove. ***Gnathochilarium***: palpi with sensory cones arranged in a single field. ***Mandibles***: not dissected. ***Stigmatic plates***: first stigmatic plate rounded, apex well-rounded, straight towards coxa 1. ***Laterotergites***: laterotergite 1 strongly elongated into a well-ronded tip. Laterotergite 2 with a broad, stout, much shorter projection. ***Collum***: with few setae on surface, anterior and posterior margins with 3–4 rows of short setae. ***Thoracic shield***: surface like tergites, longer setae only in grooves. Slope towards groove without anterior, but with 3 or 4 posterior keels. ***Tergites***: surface densely setose with short setae standing in pits. Tips of paratergites of midbody tergites slightly projecting posteriorly. ***Endotergum***: inner section lacking any spines or setae. Middle area with a single row of large, sparse, elliptical, cuticular impressions. Distance between impressions >2× their diameter. Apically, two sparse rows of marginal bristles, tips of longest setae slightly protruding beyond tergal margin (Fig. 3B). Bristles not smooth, but barbed, with numerous small spinicles. ***Anal shield***: large, in both sexes regularly rounded. Surface in both sexes completely covered with tiny setae located in small pits. Underside with a single, long, black, locking carina 2× as long as width of last laterotergite. Carina located close to last laterotergite. ***Legs***: leg 1 with 6, leg 2 with 7, leg 3 with 7 or 8 ventral spines. First two leg-pairs without an apical spine. Leg-pairs 4–21 with 10–12 ventral spines and one dorso-apical spine. In leg 9, femur 1.6, tarsus 4.5 times longer than wide (Fig. 6F). All podomeres densely setose. Coxa with a large and process dentate at margins. Coxa process absent from first and sharply projecting in second leg. Prefemur at apical margin with a projection mesally. Femur extended mesally into a dentate margin featuring 12–14 teeth. ***Female sexual characters***: vulva large, covering half of coxa, extending mesally to anterior third of prefemur (Fig. 6H). Operculum rounded, very slightly invaginated medially, mesal margin slightly projecting into a well-rounded lobe slightly higher than remaining operculum. ***Subanal plate***: large and wide, centrally recessed. ***Male sexual characters***: gonopore covered with a single, undivided, circular, sclerotized plate. ***Anterior telopods*** (Fig. 6C–E): consisting of 4 telopoditomeres distal to syncoxite. Telopoditomere 1 rectangular, as long as wide. Telopoditomere 2 large, without process, as long as telopoditomere 1. Process of telopoditomere 2 located posteriorly, visible mesally in anterior view. Process wide, well-rounded, projecting to basal part of telopoditomere 3. Telopoditomeres 3 and 4 slightly curved mesally. Telopoditomere 3 small, cylindrical, slightly shorter than telopoditomere 4, with a spine juxtaposed to process of telopoditomere 2. Telopoditomere 4 cylindrical, well-rounded, posterior side with 2–4 small spines. All telopoditomeres covered with long setae. ***Posterior telopods*** (Fig. 6A, B): telopoditomere 1 narrow, as long as wide. Immovable finger (process of telopoditomere 2) slightly shorter than movable finger, consisting of telopoditomeres 3 and 4. Immovable finger with a characteristic, distally swollen apex, clearly rounded apically, apex only slightly wider than base. Swelling only slightly expressed basally. Telopoditomere 3 rectangular, well-rounded, apically with a clearly rounded extension carrying three small spines. Telopoditomere 4 only slightly shorter and much more slender than telopoditomere 3, 5.2× as long as wide, apically weakly tapering, straight, not curved, with 23 small spines at margin towards immovable finger close to tip. In anterior view, telopoditomeres 1–3 covered with setae, in posterior view telopoditomeres 2–4 more glabrous except for numerous setae at margins.

**Figure 4.**
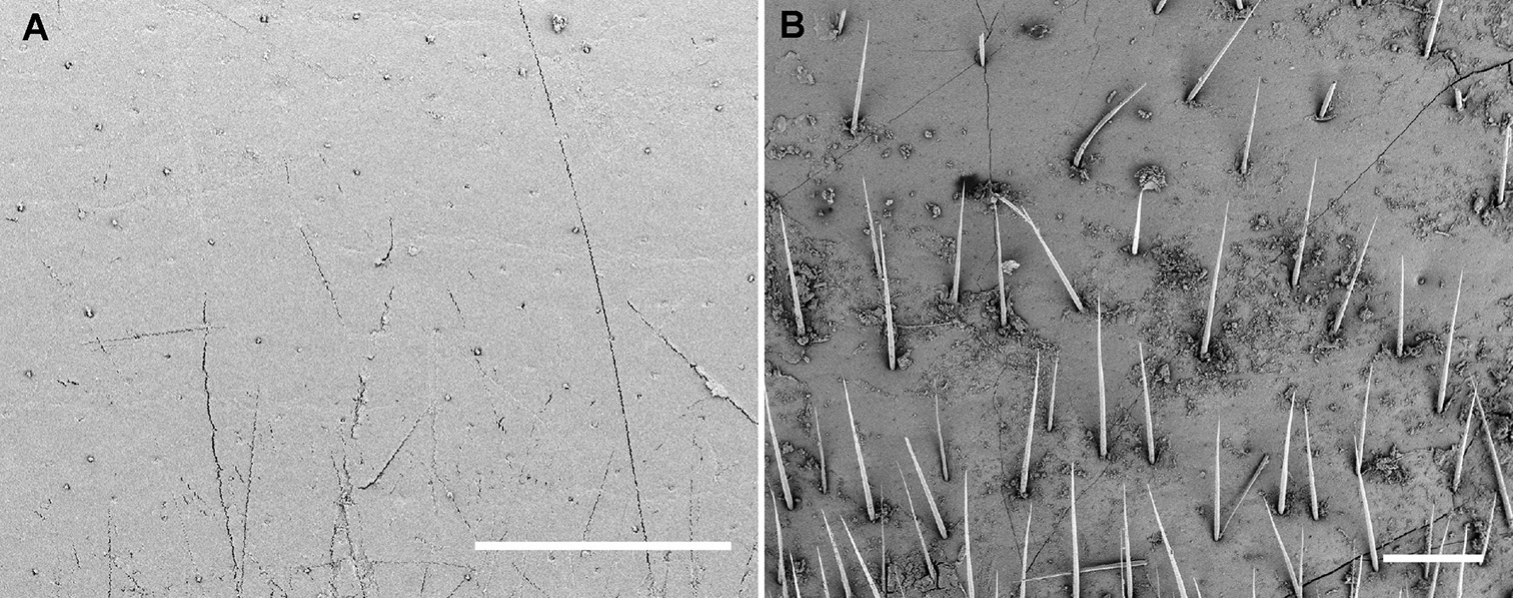
Tergal surface of midbody segments, SEM micrographs. **A***Sphaerobelum bicorne* Attems, 1938, ♂ (ZFMK) **B***Sphaeropoeus bidoupensis* sp. nov., ♂ holotype. Scale bars: 0.2 mm.

##### Etymology.

To emphasize the provenance from the Pu Mat National Park, adjective.

##### Remarks.

In the field, the weather was fluctuating between very dry and hot days and several rainy days, with day temperature above the leaf litter averaging 24 °C, and night temperature averaging 21 °C, not dropping below 18.5 °C. *Sphaerobelum pumatense* were quite rare in any weather conditions. The millipedes were found in forests ranging from 150 m to 400 m a.s.l., the forest canopy appeared to have been free of this species. Females were mostly hidden in leaf litter in small patches of litter on very steep slopes (60–70°), forming “living rooms”, choosing places with water oozing from beneath shale so that the leaf litter was constantly wet. Males were found mainly on the forest floor at night. No juveniles were seen.

**Figure 5.**
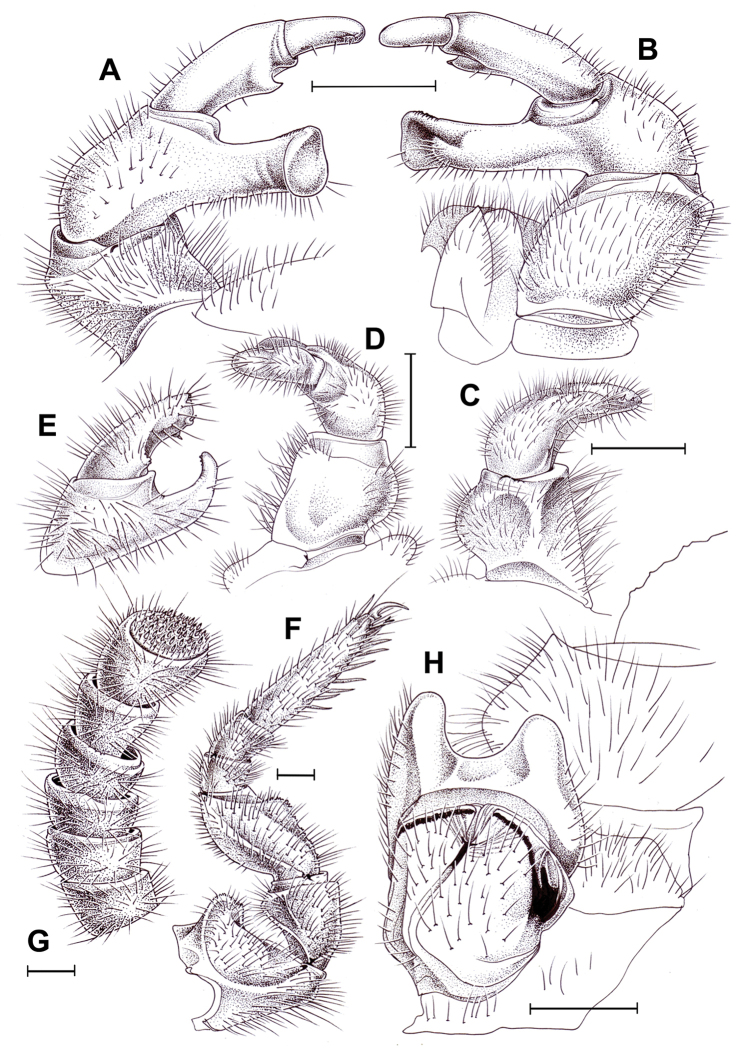
*Sphaerobelum bicorne* Attems, 1938, ♂ (**A–G**) and ♀ (**H**) from Song Thanh National Park. **A, B** Right posterior telopod, anterior and posterior views, respectively **C, D** right anterior telopod, posterior and anterior views, respectively **E** telopoditomeres 2 and 3 of right anterior telopod, lateral view **F** left leg 9, anterior view **G** left antenna **H** left coxa and prefemur 2 with vulva, posterior view. Scale bars: 1.0 mm, **E** drawn not to scale.

### Genus *Sphaeropoeus* Brandt, 1833

See Wesener (2016b) for a redescription, a diagnosis and a phylogenetic analysis of the genus.

**Remarks.** A large gap between the tarsal claw and apical spine as observed in the three species of *Sphaeropoeus* studied here, as well as in the two (including the type species *S. hercules* Brandt, 1833) redescribed recently (Wesener 2016b); this may represent another feature characteristic of the genus. Another leg character that is conspicuous in all five recently studied species of the genus is the very long femoral ridge.

#### Sphaeropoeus manca

Taxon classificationAnimaliaDiplopodaZephroniidae

(Attems, 1936) comb. nov.

B7828946-7716-57F1-84F8-A39DB9E01C26

Zephronia manca Attems, 1936: 169; Sundara 1970: 127 (list); Golovatch 1983 (list); Enghoff et al. 2004: 32 (list); Jeekel 2001: 20 (list); Wesener 2016b: 33 (list).

##### Syntypes.

♂ and ♀, NHMW 2239 (not examined).

##### Distribution.

Vietnam: D’Ran, Lam Dong Prov., near Dalat; Peak Lang Biang, Lam Dong Prov., S. Annam. India: Dhobie Jhora, Kurseong, eastern Himalayas (Attems 1936).

**Figure 6.**
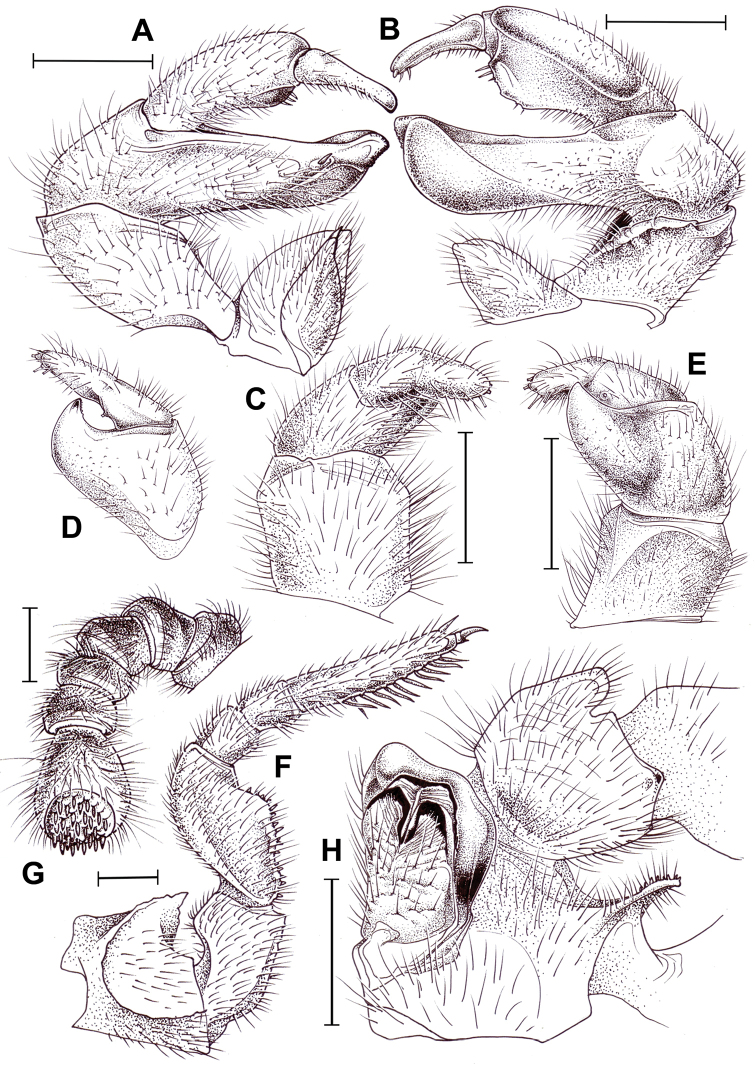
*Sphaerobelum pumatense* sp. nov., ♂ holotype (**A–G**) and ♀ paratype (**H**). **A, B** Left posterior telopod, anterior and posterior views, respectively **C–E** left anterior telopod, anterior, lateral and posterior views, respectively **F** left leg 9, anterior view **G** left antenna **H** left coxa and prefemur 2 with vulva, posterior view. Scale bars: 1.0 mm, **D** drawn not to scale.

##### Remarks.

The drawings of the telopods, as well as the extremely enlarged operculum of the ♀ vulva as depicted in the original description (Attems 1936) clearly show this species to be a member of the genus *Sphaeropoeus*, related to the other species mentioned below. Only a full revision of the type series, which contains syntypes from several localities in Vietnam and India, may clarify whether several species are actually hidden under the name *S. manca*. The posterior telopods and other characters are clearly different in *S. manca* compared to the two other species, as well as to *Sphaeropoeus maculatus* (Verhoeff, 1924) which is redescribed below.

#### Sphaeropoeus maculatus

Taxon classificationAnimaliaDiplopodaZephroniidae

(Verhoeff, 1924)

65370F4F-B32A-5046-8907-6B09D302690B

Figs 3C , 7 , 10

Tonkinobelum maculatum Verhoeff, 1924: 62; Attems 1936: 192 (list); Wang 1967: 484 (list); Korsós 1983: 118 (list); Golovatch 1983 (list)
Sphaeropoeus maculatus ―Jeekel 2001: 23 (list); Enghoff et al. 2004: 32 (list); Wesener 2016a: 40 (list); Wesener 2016b: 146 (list).

##### Material examined.

***Lectotype*** ♂ (HNMB 2858/1), designated here to fix the name for future studies and to avoid taxonomic confusion. Vietnam, ‘Tonkin’, Mau Son (= Mau Son Mountains, Lang Son Province), H. Fruhstorfer leg.

##### Redescription.

**Figure 7.**
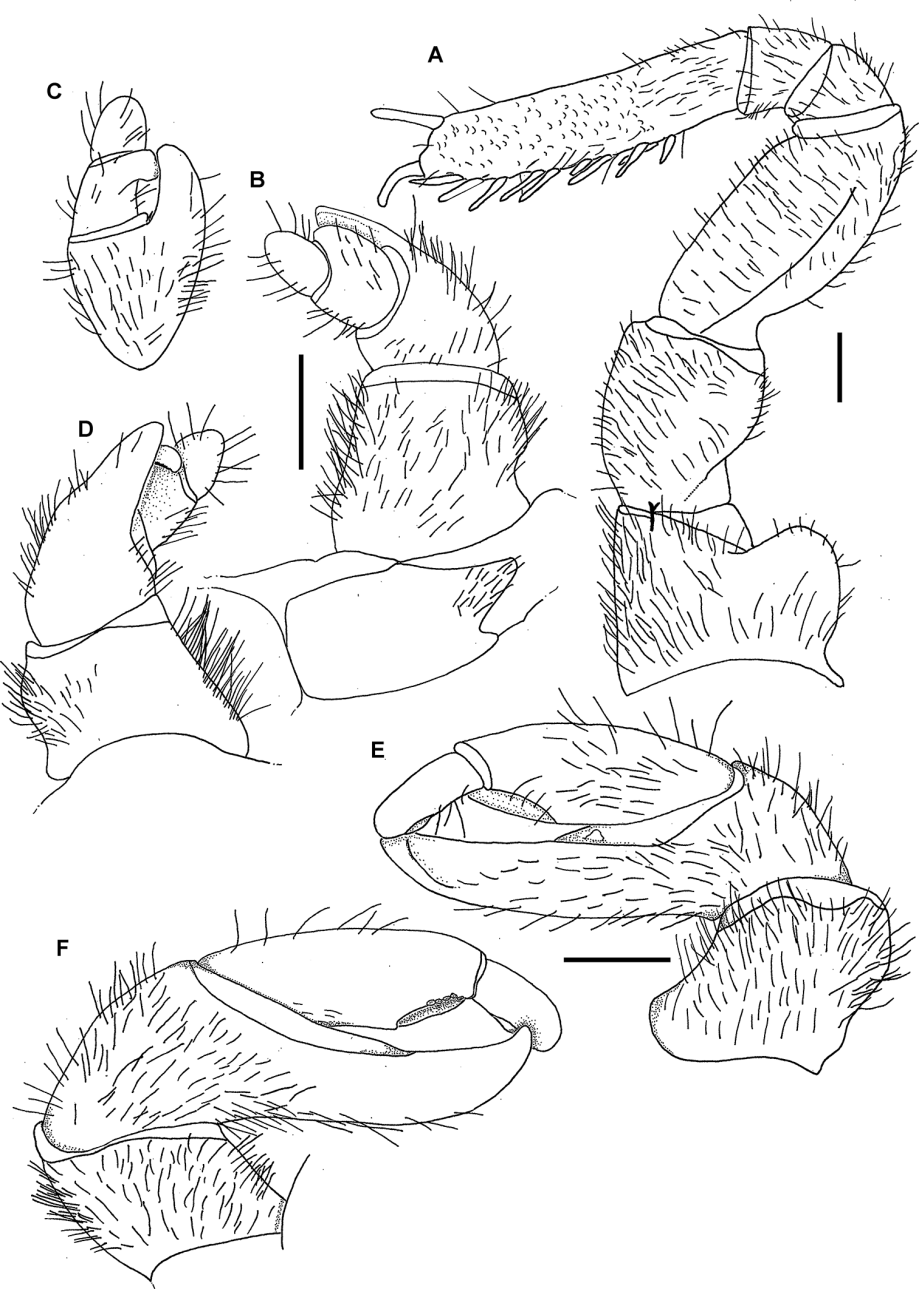
*Sphaeropoeus maculatus* (Verhoeff, 1924), ♂ lectotype. **A** Left leg 9, posterior view **B–D** right anterior telopod, anterior, lateral and posterior views, respectively **E, F** right posterior telopod, anterior and posterior views, respectively. Scale bars: 1.0 mm.

***Measurements***: ca. 58 mm long, 23.1 mm (2^nd^), up to 23.9 mm (8^th^) wide, 13.5 mm (2^nd^) up to 15.1 mm height (8^th^ the highest). ***Coloration***: apparently faded after more than 90 years in ethanol. Head, collum and appendages dark green, remaining tergites castaneous brown. ***Head***: eyes with >70 ocelli. Antennae very short, protruding up to centre of head. Antennomeres 1–5 with few longer setae, 6^th^ densely pubescent. Antennomere 6 towards disc with a single row of sensilla basiconica. Antennomere 6 strongly axe-shaped, twice as wide as antennomeres 1–5. ♂ with >140 apical cones. Palpi of gnathochilarium located in a single field. ***Collum***: glabrous except for anterior edges. ***Thoracic shield***: with wide and deep grooves, 3 or 4 weak crests present at posterior corner. ***Tergites***: surface glabrous and smooth except for paratergite depressions. Midbody paratergite tips projecting posteriorly. ***Anal shield***: well-rounded, glabrous. Locking carinae rudimentary, very short, located close to last laterotergite. ***Endotergum***: inner section lacking any spines or setae. Middle area lacking discernible cuticular impressions. Apically, 3–4 very dense rows of short marginal bristles, tips of longest setae barely protruding beyond midpoint towards tergal margin (Fig. 3C). Bristles not smooth, but barbed, with numerous small spinicles. ***Stigmatic plates***: first well-rounded, not triangular in shape. ***Laterotergites***: first elongated into a strongly tapering sharp process. Laterotergite 2 also extended, with a very sharp tip. Laterotergites 3 and following not extended, well-rounded. ***Legs***: first with 2, second with 5, third with 8 ventral and a single apical spine. Leg-pairs 4–21 with 10–12 ventral spines. Coxa process visible, well-rounded (Fig. 7A). Femur 2.1, tarsus 4 times longer than wide. Femur with a very long ridge (Fig. 7A). ***Male gonopore***: opening covered with a single, apically membranous plate. ***Anterior telopod*** (Fig. 7B–D): four podomeres, first two of equal length, 3^rd^ half as long as 2^nd^ discarding its process, 4^th^ slightly shorter and more slender than 3^rd^. Telopoditomere 2 with a strong, curved process. Telopoditomere 3 posteriorly with a shorter process juxtaposed to apex process of telopoditomere 2. Telopoditomere 4 conical, lacking any spines, lobes or teeth. ***Posterior telopod*** (Fig. 7E, F): podomeres 3 and 4 longer than process of podomere 2. Podomere 4 short, conical, with a membranous ledge and no visible spines. Podomere 3, 2.8 times longer than wide. Its excavated inner margin with a membranous ledge, in posterior aspect with ca. 4 small crenulated teeth. Immovable finger wide, apically tapering, tip curved towards movable finger. Membranous area with at least one large membranous lobe. Podomeres 1–3 in anterior view with numerous setae, in posterior view telopoditomere 3 with few setae mainly located at margins. Podomere 4 in both aspects glabrous, except for 3 or 4 long setae at mesal margin.

##### Remarks.

Verhoeff described the species based on four syntypes. Only one syntype could be relocated; the others may be considered as likely lost. As the sole available syntype is a mature ♂ in very good condition, this specimen has been designated as the lectotype. ♀ unknown.

Jeekel (2001) thought the specimen had been mislabeled, because no other species of the genus was then known from Vietnam or continental Asia north of Singapore. However, with the new combination of *Zephronia manca* (see above), and the two new species described below, the provenance of *S. maculatus* may well be correct.

#### Sphaeropoeus honbaensis sp. nov.

Taxon classificationAnimaliaDiplopodaZephroniidae

4FCE2905-06CA-53B5-B8B7-8EFAD5029C4F

http://zoobank.org/8C6B0566-6BEE-4A06-B59F-BDA14A90892B

Figs 2F–H , 3E , 8

##### Material examined.

***Holotype*** ♂ (ZMUM Rd 4644), Vietnam, Khanh Hoa Prov., Hon Ba Nature Reserve, 12°07'N, 108°56'E, 1550 m a.s.l., mixed mossy tropical forest on mountain ridge, on forest floor, night time, VI.2018, I.I. Semenyuk leg. ***Paratypes*** 2 ♂ (ZMUM Rd 4633), 1 ♂ (ZFMK MYR8943), same locality as holotype. 1 ♀ (ZMUM Rd 4645), same locality, 1450 m a.s.l., mixed tropical forest on mountain slope, in leaf litter, day time, VI.2018, I.I. Semenyuk leg.

**Figure 8.**
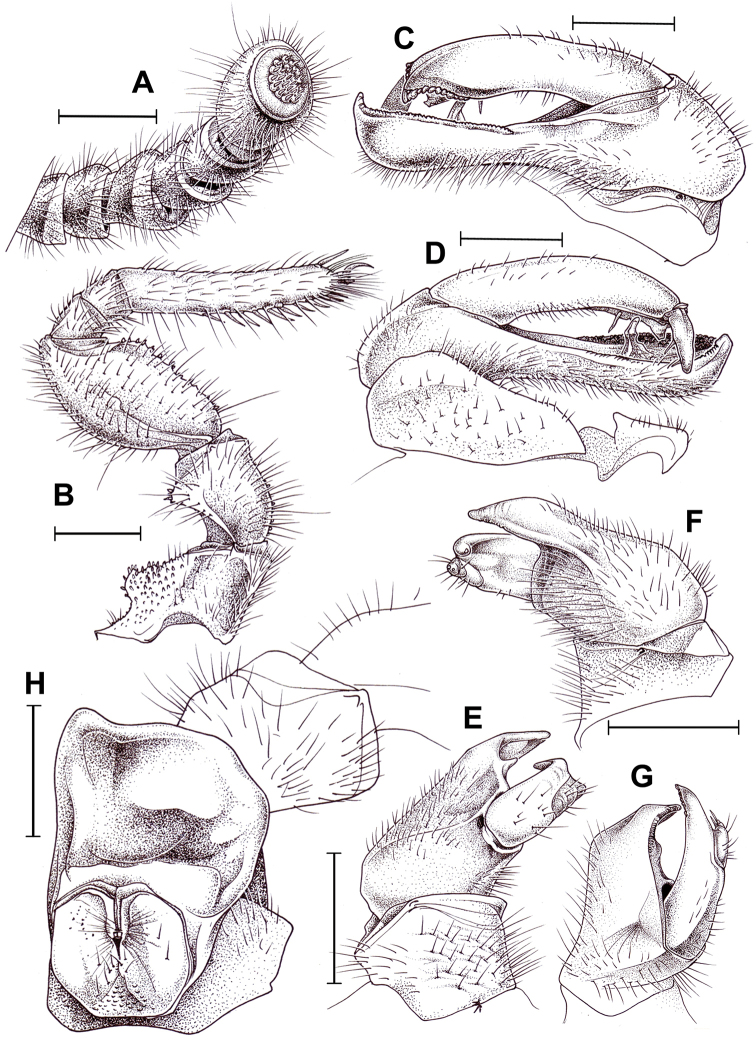
*Sphaeropoeus honbaensis* sp. nov., ♂ holotype (**A–G**) and ♀ paratype (**H**). **A** Left antenna, **B** left leg 9, anterior view **C, D** left posterior telopod, posterior and anterior views, respectively **E–G** left anterior telopod, anterior, posterior and lateral views, respectively **H** left coxa and prefemur 2 with vulva, posterior view. Scale bars: 1.0 mm, **G** drawn not to scale.

##### Diagnosis.

*Sphaeropoeus honbaensis* sp. nov. differs from all other known continental species of the genus in the presence of very few (< 30) apical cones on the ♂ antenna (usually at least >70, often >120). The anterior telopod also shows a very short, almost completely reduced telopoditomere 4, a character only shared with *S. bidoupensis* sp. nov. *Sphaeropoeus honbaensis* sp. nov. differs from *S. bidoupensis* sp. nov. in the lack of a spine in the inner area of the large telopoditomere 3 (vs. present in *S. bidoupensis* sp. nov.), in the endotergum, where the distances between the cuticular impressions are wider than the diameter (vs. slightly narrower than diameter in *S. bidoupensis* sp. nov.), the ♂ anal shield being weakly bell-shaped (vs. well-rounded in *S. bidoupensis* sp. nov.), and in leg structure, with leg-pair 3 lacking an apical spine (vs. present in *S. bidoupensis* sp. nov.), the prefemur lacking a dentate mesal margin (vs. present in *S. bidoupensis* sp. nov.), and the coxa process being strongly developed and well-rounded (vs. weakly developed and partly sharp in *S. bidoupensis* sp. nov.).

##### Description.

***Measurements***: holotype ♂ ca. 27 mm long, 12.1 mm (2^nd^), up to 12.2 mm (7^th^) wide, 7.8 mm (2^nd^) up to 15.1 mm height (8^th^ the highest); ♂ paratypes 8–11 mm wide. Paratype ♀ ca. 29 mm long, 13.1 mm (2^nd^), up to 13.3 mm (7^th^) wide, 7.8 mm (2^nd^) up to 8.4 mm height (7^th^ the highest). ***Coloration***: both in vivo and in vitro, after >1.5 years of preservation in ethanol, similar, in life uniformly dark violet brown to violet blackish with vague infuscate bands near caudal margin (Fig. 2F–H), in alcohol dark brown to brown, in places marbled, only dorsalmost part of anal shield sometimes lighter centrally, light brown. Antennae orange. Legs mostly grey- or olive-brown, but tarsi yellow-brown. Tegument mosly dull to poorly shining. ***Head***: eyes with ca. 65 ocelli. Antennae short (Fig. 8A), protruding beyond centre of head. Antennomeres 1–5 with few longer setae, 6^th^ densely pubescent. Antennomere 6 towards disc with single row of sensilla basiconica. Antennomere 6 slightly swollen in ♂, cylindrical in ♀, twice as long as, but only slightly wider than, antennomeres 1–5. ♂ with 26/27, ♀ with 22/24 apical cones. Palpi of gnathochilarium located in a single field. ***Collum***: completely covered with long setae, like the tergites. ***Thoracic shield***: with wide and shallow grooves, 3 or 4 weak crests present at posterior corner. ***Tergites***: surface covered with longer setae, most innervating in small pits. Midbody paratergite tips projecting posteriorly (Fig. 10). ***Anal shield***: well-rounded in ♀, weakly bell-shaped in ♂. In both sexes completely covered with longer setae. Locking carina long, twice as long as width of last laterotergite, located close to margin. ***Endotergum***: inner section lacking any spines or setae. Middle area with a single row of large, sparse, elliptical, cuticular impressions. Distance between impressions greater than their diameter. Apically, two rows of long marginal bristles, tips of longest setae clearly protruding beyond tergal margin (Fig. 3E). Bristles not smooth, but with numerous small spinicles. ***Stigmatic plates***: first well-rounded, triangular. ***Laterotergites***: first with a slightly projecting, well-rounded process. Laterotergites 2 and following not extended, well-rounded. ***Legs***: first with 2 or 3, second with 3 or 4, third with 8 ventral and lacking an apical spine. Leg-pairs 4–21 each with 10–12 ventral spines and a single apical spine. Coxa process visible, well-rounded (Fig. 8B). Femur 1.7, tarsus 5.2× as long as wide. Femur with a very long ridge (Fig. 8B). Mesal margin of femur completely extended into 12–14 teeth, lateral margin of prefemur, juxtaposed to coxal process, slightly extended into 2–4 teeth (Fig. 8B). ***Female sexual characters***: vulva large, covering 2/3 coxa, a conspicuous operculum extending to basal half of prefemur (Fig. 8H). Operculum massive, larger than bursa, wider than prefemur, apically rounded, mesal margin very slightly projecting and slightly higher than remaining operculum. ***Subanal plate***: large and wide, triangular. ***Male gonopore***: opening covered with a single, apically membranous plate. ***Anterior telopod*** (Fig. 8E–G): four podomeres, first three of equal length regardless of the processes, podomere 4 rudimentary, conical. Telopoditomere 2 with a strong, curved process overreaching telopoditomere 4. Telopoditomere 3 posteriorly with a longer process juxtaposed to apex process of telopoditomere 2, clearly protruding above telopoditomere 4, as well as process of telopoditomere 2. Telopoditomere 4 conical, with two spines. ***Posterior telopod*** (Fig. 8C, D): Podomeres 3 and 4 slightly longer than process of podomere 2. Podomere 4 short, conical, with two spines, slightly curved towards immovable finger. Podomere 3 slender, 4.2 times longer than wide. Its excavate inner margin with a membranous lobe and a single spine, posterior aspect with ca. 12 small crenulated teeth. Immovable finger slender, apically tapering, tip curved towards movable finger. Membranous area apically with a large membranous lobe. Podomeres 1–3 in anterior and posterior views with few setae. Podomere 4 in both aspects glabrous.

**Figure 9.**
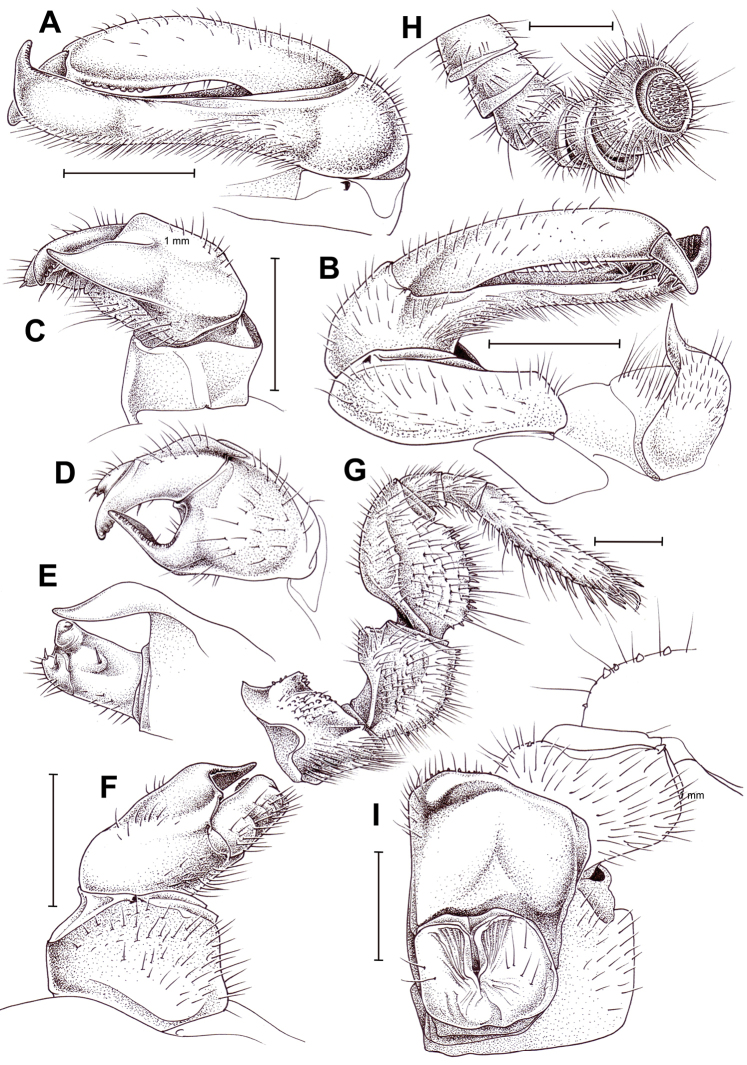
*Sphaeropoeus bidoupensis* sp. nov., ♂ holotype (**A–H**) and ♀ paratype (**I**). **A, B** Left posterior telopod, posterior and anterior views, respectively **C–F** left anterior telopod, posterior, sublateral, lateral and anterior views, respectively **G** left leg 9, anterior view **H** left antenna **I** left coxa and prefemur 2 with vulva, posterior view. Scale bars: 1.0 mm, **D** drawn not to scale.

##### Etymology.

To emphasize the provenance from the Hon Ba Nature Reserve, adjective.

##### Remarks.

During the expedition, more than ten males of this species were recorded, all walking on the forest floor in the night, mainly on the mountain ridge. Only one adult female was found despite special searching efforts: it was hidden in leaf litter, which is typical of sphaerotheriidans. The female was encountered in a forest at an elevation considerably lower than the where abundant males were observed. Although confusing, this may be accounted for by high population abundance at the onset of a season. During the expedition, it was raining almost every day, the temperature in the daytime above leaf litter averaging 20 °C and dropping down to 17.5 °C (minimum 16.8 °C) at night. Fog was very often seen on the top of the mountain. No juveniles were recorded.

#### Sphaeropoeus bidoupensis sp. nov.

Taxon classificationAnimaliaDiplopodaZephroniidae

461037CE-AC1A-5D90-9D24-1759F9FAA7C7

http://zoobank.org/F68EE25F-CEF3-4816-B94C-D5050AE5B5C5

Figs 2A–E , 3D , 4B , 9

##### Material examined.

***Holotype*** ♂ (ZMUM Rd 4646), Vietnam, Lam Dong Prov., Bidoup Nui Ba National Park, 12°10'N, 108°41'E, 1500 m a.s.l., mixed tropical forest on hills, in leaf litter, daytime, VI.2018, I.I. Semenyuk leg. ***Paratypes*** 1 ♂, 2 ♀ (ZMUM Rd 4634), 1 ♂ (ZFMK MYR8944), 1 ♀ (ZFMK MYR8859), same data as holotype.

##### Diagnosis.

*Sphaeropoeus bidoupensis* sp. nov. differs from almost all other known continental species of the genus in the anterior telopod showing a very short, almost completely reduced telopoditomere 4, a character only shared with *S. honbaensis* sp. nov. *Sphaeropoeus bidoupensis* sp. nov. differs from *S. honbaensis* sp. nov. in the presence of a spine in the inner area of the large telopoditomere 3 (vs. absent in *S. honbaensis* sp. nov.), in the endotergum, where the distances between the cuticular impressions are slightly smaller than the diameter (vs. wider than diameter in *S. honbaensis* sp. nov.), the ♂ anal shield being well-rounded (weakly bell-shaped in *S. honbaensis* sp. nov.), and in leg structure, with leg-pair 3 having an apical spine (vs. absent in *S. honbaensis* sp. nov.), the prefemur showing a dentate mesal margin (vs. smooth in *S. honbaensis* sp. nov.), and the coxa process being weakly developed and partly sharp (vs. strongly developed and well-rounded in *S. honbaensis* sp. nov.).

##### Description.

***Measurements***: holotype ♂ ca. 24 mm long, 11.2 mm (2^nd^), up to 12.3 mm (7^th^) wide, 7.1 mm high (2^nd^ the highest). Paratype ♀ (ZFMK): ca. 29 mm long, 13.5 mm (2^nd^), up to 14.3 mm (7^th^) wide, 7.7 mm (2^nd^) up to 9.1 mm in height (7^th^ the highest). ZMUM paratypes 10 mm (♂), 11 mm (♀) or 13 mm wide (♀). ***Coloration***: both in vivo and in vitro, after >1.5 years of preservation in ethanol, similar; in life, adults uniformly dark brown to blackish brown, juveniles lighter and showing vague or clear variegated tergal patterns (Fig. 2C, D), antennae in adults orange, legs in adults mostly dark or lighter olive-brown (Fig. 2B); in alcohol, adults likewise uniformly dark brown to blackish brown, antennae orange, legs light grey-brown to olive-grey-brown with a little lighter tarsi. Tegument mostly dull to poorly shining (Fig. 2A, B). ***Head***: eyes with ca. 65 ocelli. Antennae short (Fig. 9H), protruding beyond centre of head. Antennomeres 1–5 with few longer setae, 6^th^ densely pubescent. Antennomere 6 towards disc with a single row of sensilla basiconica. Antennomere 6 swollen in ♂, cylindrical in ♀, twice as long as, but only slightly wider than, antennomeres 1–5. ♂ with 36/40, ♀ with 17/24 apical cones. Palpi of gnathochilarium located in a single field. ***Collum***: completely covered with long setae, like the tergites. ***Thoracic shield***: with wide and shallow grooves, 3 or 4 weak crests present at posterior corner. ***Tergites***: surface covered with longer setae, most innervating in small pits (Fig. 4B). Paratergite tips of midbody tergites projecting posteriorly (Fig. 2A). ***Anal shield***: well-rounded. In both sexes completely covered with longer setae. Locking carina long, 2× as long as width of last laterotergite, located close to margin. ***Endotergum***: inner section lacking any spines or setae. Middle area with a single row of large, sparse, elliptical, cuticular impressions. Distance between impressions shorter than their diameter. Apically, two rows of long marginal bristles, tips of longest setae clearly protruding beyond tergal margin (Fig. 3D). Bristles not smooth, but with numerous small spinicles. ***Stigmatic plates***: first well-rounded, triangular. ***Laterotergites***: first with a slightly projecting, well-rounded process. Laterotergites 2 and following not extended, well-rounded. ***Legs***: first with 2 or 3, second with 5 (one of them basal), third with 8 ventral and an apical spine. Leg-pairs 4–21 each with 12–14 ventral spines and a single apical spine. Coxa process visible, partly sharp (Fig. 9G). Femur 1.5, tarsus 4.7 times longer than wide. Femur with a very long ridge (Fig. 9G). Mesal margin of femur completely extended into 12–14 teeth, prefemur at mesal margin with 5–8 teeth (Fig. 9G). ***Female sexual characters***: vulva large, covering 2/3 coxa, a conspicuous operculum extending above basal half of prefemur (Fig. 9I). Operculum massive, larger than bursa, wider than prefemur, apically rounded. ***Subanal plate***: large and wide, triangular. ***Male gonopore***: opening covered with a single, apically membranous plate. ***Anterior telopod*** (Fig. 9C–F): four podomeres, first three of equal length regardless of the processes, podomere 4 rudimentary, conical. Telopoditomere 2 with a strong, curved process overreaching telopoditomere 4. Telopoditomere 3 posteriorly with a longer process juxtaposed to apex process of telopoditomere 2, clearly protruding above telopoditomere 4, as well as both process of telopoditomere 2 and a large spine in the central area. Telopoditomere 4 conical, with a single spine. ***Posterior telopod*** (Fig. 9A, B): podomeres 3 and 4 slightly longer than process of podomere 2. Podomere 4 short, conical, with two spines, slightly curved towards immovable finger. Podomere 3 slender, 3.6 times longer than wide. Its excavate inner margin with a membranous lobe and a single spine, posterior face with ca. 10 small crenulated teeth. Immovable finger slender, apically tapering, tip curved towards movable finger. Membranous area apically with a large, bifid, membranous lobe. Podomeres 1–3 in anterior and posterior views with few setae. Podomere 4 in both aspects glabrous.

**Figure 10.**
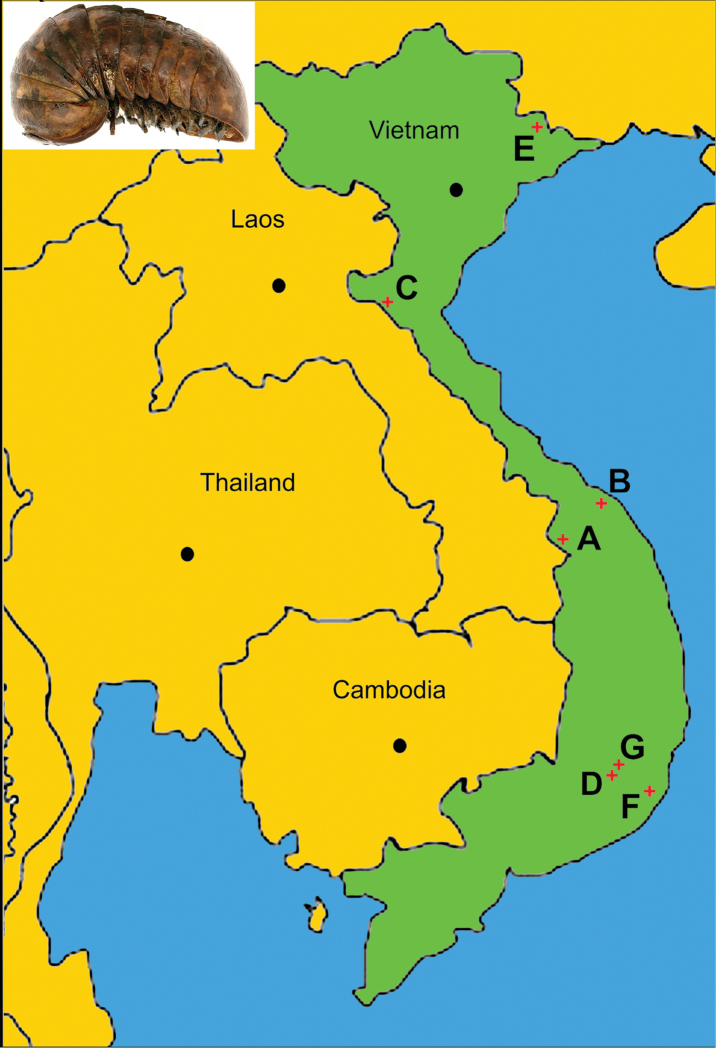
Habitus photograph of the lectotype (HNHM 2858/1) of *Sphaeropoeus maculatus* (Verhoeff, 1924), lateral view, and a distribution map of all other relevant zephroniid species in Vietnam. **A***Sphaerobelum bicorne* Attems, 1938 (new record) **B***Sphaerobelum bicorne* Attems, 1938 (old record) **C***Sphaerobelum pumatense* sp. nov. **D***Sphaeropoeus manca* (Attems, 1936) **E***Sphaeropoeus maculatus* (Verhoeff, 1924) **F***Sphaeropoeus honbaensis* sp. nov. **G***Sphaeropoeus bidoupensis* sp. nov.

##### Etymology.

To emphasize the provenance from the Bidoup Nui Ba National Park, adjective.

##### Remarks.

This new species was very abundant in the Park area and could be found almost throughout the year. In January, juveniles lived under logs, but no adults were recorded. The daytime temperature above the leaf litter averaged 17 °C, dropping down to 14 °C (minimum 12.5 °C) at night; rains were quite abundant. In June, juveniles colonized decaying wood, leaf litter, suspended soils in *Asplenium* sp. ferns, and spaces under logs. Adults lived in leaf litter and the suspended soil of ferns, only occasionally and only males walking openly on the forest floor. Juveniles and some adults were often recorded hiding inside their “living chambers” (Fig. 2C, D), just like those observed in *Sphaerobelum bicorne* (see above). The daytime temperature above the leaf litter averaged 20 °C, compared to 16.5 °C (minimum 14.9 °C) at night; rains were likewise quite abundant. In November, the millipedes were mainly hidden in leaf litter. The daytime temperature above the leaf litter averaged 22 °C, vs. 14.5 °C (minimum 11.2 °C) at night; rains were particularly heavy, as a typhoon came in.

*Sphaeropoeus bidoupensis* sp. nov. seems only to occur in forests at about 1500 m a.s.l.. This was a riparian, very wet, broadleaved forest with abundant *Asplenium* sp. ferns on tree trunks starting from the ground level and a thick leaf litter layer on the floor, as well as a forest with dominating Fagaceae trees mixed with several coniferous species on slopes, the leaf litter layer being thick and to a significant proportion formed by coniferous needles. The species was not located in the adjacent mossy elfin forest up to 2000 m a.s.l. with a much cooler and wet weather.

## Conclusions

Zephroniidae in Vietnam, currently amounting to seven species of *Sphaerobelum*, five species in *Zephronia*, four in *Sphaeropoeus*, three in *Prionobelum* and one species in *Cryxus*, are distributed in the north as well as the south of the country (Fig. 10). They tend to show a remarkable pattern of each species being very narrowly endemic and mostly confined to a single locality, although more inventories in numerous unexplored areas need to be conducted. This has been observed in Laos (Wesener 2019) and Thailand (Wongthamwanich et al. 2012). In Vietnam, examples of syntopically coexisting species or even genera are likewise very few, e.g., *Sphaerobelum konkakinhense* Semenyuk, Golovatch & Wesener, 2018, *Zephronia konkakinhensis* Semenyuk, Golovatch & Wesener, 2018 and *Z. montis* Semenyuk, Golovatch & Wesener, 2018 in the montane forests of the Kon Ka Kinh National Park, Gia Lai Province or *Cryxus ovalis* Leach, 1814, *Sphaerobelum cattiense* Semenyuk, Golovatch & Wesener, 2018 and *Zephronia ovalis* Gray, 1832 in the monsoon lowland forest patch of the Nam Cat Tien National Park, Dong Nai Province, both in southern Vietnam (Golovatch et al. 2012, Semenyuk et al. 2018).

Our contribution reinforces the impression that Vietnam, together with the adjacent parts of Laos, represents one of the main hotspots of zephroniid/sphaerotheriidan diversity not only in Indochina, but also in the entire Southeast Asia. Despite the considerable recent progress achieved in the study of Sphaerotheriida in Indochina (Golovatch et al. 2012, Wesener 2016a, 2016b, 2019, Semenyuk et al. 2018), there can be little doubt that future investigations will reveal many more new species and records of giant pill-millipedes in the region. Revisionary work remains topical as well.

## Supplementary Material

XML Treatment for Sphaerobelum bicorne

XML Treatment for Sphaerobelum pumatense

XML Treatment for Sphaeropoeus manca

XML Treatment for Sphaeropoeus maculatus

XML Treatment for Sphaeropoeus honbaensis

XML Treatment for Sphaeropoeus bidoupensis
